# Strategies for Metagenomic-Guided Whole-Community Proteomics of Complex Microbial Environments

**DOI:** 10.1371/journal.pone.0027173

**Published:** 2011-11-23

**Authors:** Brandi L. Cantarel, Alison R. Erickson, Nathan C. VerBerkmoes, Brian K. Erickson, Patricia A. Carey, Chongle Pan, Manesh Shah, Emmanuel F. Mongodin, Janet K. Jansson, Claire M. Fraser-Liggett, Robert L. Hettich

**Affiliations:** 1 Institute for Genome Sciences, University of Maryland School of Medicine, Baltimore, Maryland, United States of America; 2 Oak Ridge National Laboratory, Chemical Sciences Division, Oak Ridge, Tennessee, United States of America; 3 Graduate School of Genome Science & Technology, University of Tennessee, Knoxville, Tennessee, United States of America; 4 Lawrence Berkeley National Laboratory, Earth Sciences Division, Department of Ecology, Berkeley, California, United States of America; University of Georgia, United States of America

## Abstract

Accurate protein identification in large-scale proteomics experiments relies upon a detailed, accurate protein catalogue, which is derived from predictions of open reading frames based on genome sequence data. Integration of mass spectrometry-based proteomics data with computational proteome predictions from environmental metagenomic sequences has been challenging because of the variable overlap between proteomic datasets and corresponding short-read nucleotide sequence data. In this study, we have benchmarked several strategies for increasing microbial peptide spectral matching in metaproteomic datasets using protein predictions generated from matched metagenomic sequences from the same human fecal samples. Additionally, we investigated the impact of mass spectrometry-based filters (high mass accuracy, delta correlation), and *de novo* peptide sequencing on the number and robustness of peptide-spectrum assignments in these complex datasets. In summary, we find that high mass accuracy peptide measurements searched against non-assembled reads from DNA sequencing of the same samples significantly increased identifiable proteins without sacrificing accuracy.

## Introduction

Key questions in environmental microbiology include: (i) what microorganisms are present in a particular environment, (ii) how are they functioning, and (iii) how does community structure and function vary in response to environmental conditions/changes? Recent technological advances have provided powerful experimental approaches to address these questions, with 16S rRNA-based taxonomic profiling providing extensive information about microbial composition, and metagenomic whole-genome shotgun (WGS) sequencing/shotgun community proteomics, or “metaproteomics,” providing insights into the composition and functional activities of microbial communities. In particular, metagenome sequencing with next-generation platforms has revolutionized the ability to measure and fully characterize the genomic repertoire in microbial communities.

In order to successfully identify peptide sequences using mass spectrometry (MS)-based proteomics methods, a relevant database of predicted genes derived from genome or metagenome sequences is necessary. Peptide identifications result from matching tandem mass spectra (MS/MS) against predicted fragmentation patterns of all possible *in silico* digested peptides using well-established programs [1], [2], [3]. Therefore, successful MS/MS sequence-database searching is critically dependent on the quality and accuracy of the metagenomic predicted sequence database.

Although traditional MS-based proteomic analyses of single bacterial isolates are well established, applying these methods to complex microbial communities can be challenging for several reasons, including the lack of deep sequence coverage and difficulty in assembling metagenomes from 454-reads. Considerable improvements in mass spectrometers and chromatography have been made over the past decade; however, the development of tools for optimizing metagenome-metaproteome sequence matching has not kept pace, especially when using the shorter sequence reads associated with next generation sequencing platforms such as the 454 pyrosequencer [4] and Illumina GAII [5].

While an increasing number of studies have developed computation methods for proteogenomics [6], [7] and begun to integrate metagenomic sequence data with proteome measurements [8], [9], [10], these studies have primarily focused on either single eukaryotic genomes or populations with low diversity, allowing for sufficient depth of sequence coverage of abundant community members that facilitate proteome identifications as compared to more complex microbial communities (e.g., human microbiome, ocean, and soil). In the human distal gut, there are approximately 1,000 estimated species which represent >7,000 prokaryotic strains; therefore, the complete metagenome is estimated to be >100 times the size of the human genome [11]. Based on previous studies of these exact same samples, we would expect ∼30% of the proteins identified by proteomics to be of human origin [12]. The challenges inherent in a metagenomic-metaproteomic characterization of complex environmental samples include (i) considerable sequence diversity among closely related strains/species, (ii) large number of organisms for which no reference genome sequence is available and (iii) low nucleotide sequence coverage for the microorganisms, especially low abundance members.

**Figure 1:**
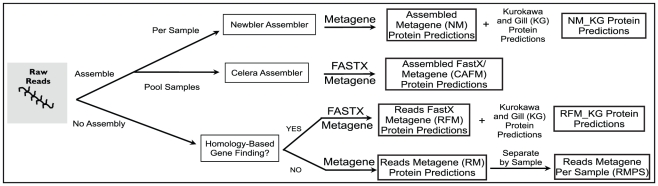
Creation of protein sequence databases. Protein sequence databases were created from metagenomic sequence reads using a variety of methods for assembly and gene finding.

Here we present a benchmarking of strategies for integration of metagenomic and metaproteomic data derived from the same human gut microbiome samples. Although the metagenomes were not sequenced to saturation, they were sufficient to enable us to evaluate how protein predictions based on metagenome data impact peptide-spectrum assignments in matched metaproteomic datasets (i.e., metagenome and metaproteome of the exact same sample). Using 454 pyrosequencing, 1,079 Mbp of DNA sequence was obtained from two fecal samples obtained from a pair of healthy twins [13]. Using these data, four protein sequence databases were created using several different assembly and gene finding strategies (Fig. 1). The resulting databases were evaluated for their utility in MS sequence-database searching.

Assembly of metagenomic reads can potentially generate errors by joining sequence reads that share sequence identity but are derived from different strains or species. This can be further complicated by sequencing errors, such as issues with homopolymer tracts in 454 pyrosequencing datasets [14], [15]. The metagenome assembly strategies examined in this study were (i) assembly by sample, exemplifying the traditional approach used for single isolate genomes, (ii) whole-dataset assembly, in order to increase sequence coverage, and (iii) no assembly, which will theoretically capture all sequence diversity present in a sample. Since sequencing errors can also introduce frameshifts and in-frame stop codons, resulting in fragmented gene predictions, we explored homology-based gene finding, as it allows the ability to ‘gap’ over sequencing errors, and *de novo* based gene finding which uses models of known gene structure for prediction.

Proteomics approaches were also benchmarked to identify the parameters necessary to create accurate peptide-spectrum matches (PSMs; a match of a given MS/MS spectrum to a specific database peptide sequence) and increase protein discovery by *de novo* peptide sequencing. Several MS-related parameters (spectral quality, delta correlation (deltCN), and high mass accuracy (±10 ppm (parts-per-million)) were examined and proved to be helpful in providing more comprehensive, confident PSMs. Moreover, we investigated how much *de novo* peptide sequencing would increase peptide identification, since it provides novel sequences that were not originally present in the sequence database (e.g., polymorphisms). By utilizing the genomic and proteomic tools described in this study, we identified a strategy that increased the number of PSMs and protein identifications in a complex microbial community that can provide a more comprehensive and accurate characterization of the human gut microbiome.

## Methods

### Samples, DNA and Protein Extraction

Fecal samples from two healthy female human individuals (a concordant twin pair), numbered 6a and 6b, were collected under a separate study, as described and studied previously [12]. Both samples were used for DNA and protein extraction. An additional three twin pairs corresponding to six human fecal samples: numbered 15a and 15b (concordant pair with Crohn's disease), 16a and 16b (discordant pair, healthy 16a and 16b with Crohn's disease), and 18a and 18b (discordant pair, healthy 18a and 18b with Crohn's disease), were used for metagenomic sequencing only and were included in several of the sequence databases as described in “Protein Database Construction.” Therefore, a total of two healthy samples (6a and 6b) were used for metaproteomics and eight (four healthy: 6a, 6b, 16b, and 18b and four diseased: 15a, 15b, 16a, 16b) samples were used for metagenomics. Throughout the manuscript, the diseased samples and individuals other than 6a and 6b are referred to as “unrelated” because we are only focusing on 6a and 6b samples' metaproteomes, thus, we have a “matched” or “related” metagenome-metaproteome. Since these fecal samples were collected under a separate research program and were supplied as de-identified information for this study, this work was approved in March 2010 by the Oak Ridge Site-wide Institutional Review Board (ORSIRB; Dr. Leigh Greeley, chair-person) as “human studies exemption 4”, *IRB REFERENCE #: ORNL EX(10)-3*.

Total genomic DNA was extracted using the MoBio PowerSoil DNA Isolation kit (MoBio Laboratories, Carlsbad, CA) following the manufacturer's recommendations. Sample 6a was also extracted using the Zymo extraction protocol recently published by Ravel and colleagues [16]. Each sample was then sequenced using Roche 454 FLX-Titanium pyrosequencing according to manufacturer specifications. Raw sequence data were processed using the Roche/454 run processing software to filter short, mixed, and low quality reads. The sequencing generated 418K −627 M passed-filter reads and 170–381 Mbp per sample for the eight human fecal samples (15a, 15b, 16a, 16b, 18a, 18b, 6b, and 6a). Microbial cells (∼100 mg cell pellet) and proteins were extracted and processed for two-dimensional liquid chromatography coupled to tandem mass spectrometry (2D-LC-MS/MS). The protocol for cell lysis and protein extraction has been rigorously tested and developed by our laboratory [17], [18] with specific details corresponding to these samples detailed in Verberkmoes *et al*. [12].

### Protein Database Construction

Starting with 454 pyrosequencing reads, four metagenomic processing methods (NM, RM, RFM, and CAFM, see below for definitions) were evaluated for the construction of predicted protein databases (Fig. 1 and Table 1). Sequences were first filtered for human contamination by alignment of reads to the human genome (v 36) using NUCMER [19] using default parameters. The Newbler-Metagene (NM) protein sequence database was created using the single-genome strategy by generation of a *de novo* assembly followed by *de novo* gene finding. While there are a variety of gene prediction algorithms available, we chose to focus on MetaGene Annotator [20], a platform that we have extensive experience with for 454 sequencing datasets. Certainly, newer approaches, such as Orphelia [21], MetaGeneMark [22], and FragGeneScan [23] have appeared and shown promise for Illumina datasets; however, the accuracy of these algorithms do not appear to differ greatly for 300–400 bp reads and thus we preferred to utilize our more rigorously tested MetaGene version.

**Table 1:** Performance and comparison of the metagenomic predicted protein sequence databases.

Metagenomic Predicted Protein Sequence Database		Celera Assembler, Fastx, Metagene	Newbler, Metagene	Newbler, Metagene + Kurokawa/Gill	Raw Reads Metagene	Raw Reads, FastX, Metagene	Raw Reads, FastX, Metagene + Kurokawa/Gill	Raw Reads, Metagene Paired Search
Database Acronym		CAFM	NM	NM_KG	RM	RFM	RFM_KG	RMPS
Number of Sequences (thousand)		1,844	190	540	1,903	1,520	1,907	2,146
Number of Amino Acids (million bp)		200	45	115	189	173	262	191
Compute Time Per Run (minutes)		670	80	320	750	1,060	1,030	435
Number of Non-redundant Spectra	6a Run 2	5,179	6,235	10,441	9,100	9,074	10,975	13,806
	6a Run 3	4,326	5,376	9,272	8,152	8,538	10,330	18,401
	6b Run 1	4,092	5,615	10,830	8,639	8,480	11,254	12,363
	6b Run 2	3,873	5,800	10,724	8,775	8,573	11,167	12,212
Total Spectra		***17,470***	***23,026***	***41,267***	***34,666***	***34,665***	***43,726***	***56,782***
Total number of PSMs within ±10 ppm		***14,317***	***16,906***	***31,289***	***26,181***	***25,997***	***33,347***	***39,681***
Number of Non-redundant Peptides	6a Run 2	4,383	3,093	5,678	4,710	4,669	5,911	7,592
	6a Run 3	3,655	2,403	4,617	3,804	3,963	5,068	6,303
	6b Run 1	3,404	2,426	5,409	3,919	3,879	5,549	5,923
	6b Run 2	3,216	2,297	5,088	3,747	3,690	5,238	5,605
Total Peptides		***14,658***	***10,219***	***20,792***	***16,180***	***16,201***	***21,766***	***25,423***
Total NR Peptides		***8,632***	***5,994***	***12,406***	***9,618***	***9,608***	***13,111***	***16,055***

The database composition and SEQUEST/DTASelect search results (compute time, identified non-redundant spectra and peptides) with a 2-peptide and deltCN of 0.08 filters are shown for samples 6a (Run 2 and 3) and 6b (Run 1 and 2).

Shotgun sequences from each sample were assembled using the Newbler Assembler (v2.0.01.14), and genes were predicted on contigs greater than 500 bp using Metagene [24], resulting in a total of 153,586 predicted open reading frames (ORFs) larger than 50 nt across a total of the seven metagenome samples included in this study. The second database, Reads-Metagene (RM), was created by directly predicting ORFs from raw sequencing reads to prevent loss of sequence diversity when collapsing unrelated sequencing reads during genome assembly. ORFs were predicted using Metagene, yielding 1,866,893 predicted ORFs larger than 50 nt. Sequencing errors often seen in pyrosequencing datasets [14], [15] can lead to artificially fragmented predicted ORFs. Because these errors cause frameshifts and in-frame stop codons in gene predictions, we used protein-to-DNA alignments, generated by sequence similarity searches against NCBI's NR using FASTX [25] with an expectation value threshold of 1e^−6^, to predict genes by homology. Homology-based gene finding was performed on raw 454 sequencing reads yielding 1,483,958 predicted ORFs larger than 50 nt, called Reads-FASTX-Metagene (RFM) protein database.

Additionally, three databases were created from assembled reads, with the intent of creating longer genes and fewer protein fragments. The combination of short sequencing reads, averaging 369 bp, and the high bacterial diversity found in the human gut, produced a dataset with many fragmented genes. Since assembled sequences were not much longer than raw sequencing reads, these genes were also fragmented, therefore, we were unable to validate proteins identified by multiple peptide matches. Thus, an assembly was created by combining the shotgun sequence data from these samples using the Celera Assembler (v5.4), called Celera Assembler-FASTX-Metagene (CAFM), yielding 1,807,963 predicted proteins on all contigs and singletons larger than 50 nt. Homology-based gene finding was also used for this CAFM database, using the same parameters as RFM. In addition to sequences generated in this study, we included the following published human gut metagenomic datasets: two metagenomes from Gill *et al*. [26] and thirteen metagenomes from Kurokawa *et al*. [27], that were concatenated with the NM (termed NM_KG) and RFM (termed RFM_KG) sequence databases to provide additional sequence variation and increase proteome coverage. The metagenomes published from Gill *et al*. [26] (17,688 contigs; ORFs ≥20 amino acids; ∼50,000 predicted proteins; available at the Joint Genome Institute (JGI) IMG database under NCBI project ID 16729) and Kurokawa *et al*. [27] (81,968 contigs; ORFs≥50 amino acids; ∼300,000 predicted proteins; available at CAMERA (2007)) studies were sequenced via Sanger-based methods. The amino acid sequence of the proteins belonging to the two samples' metagenomes used in this study (6a and 6b in addition to 15a, 15b, 16a, 16b, 18a, 18b) can be accessed through the NCBI Protein Database under NCBI project ID 46321.

**Figure 2:**
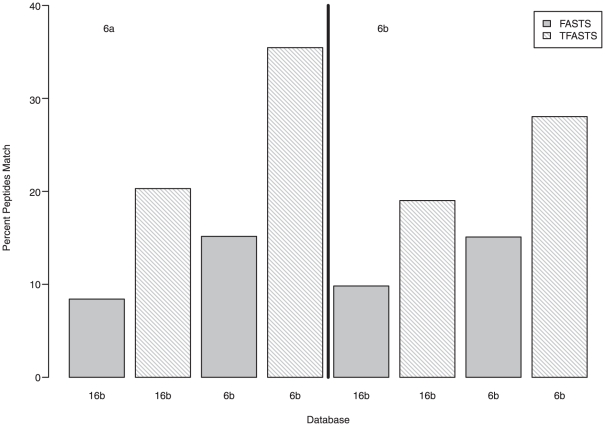
Comparison of identified peptides using sequence similarity techniques. Percentage of matches found when comparing identified peptides from sample 6a (left panel) or 6b (right panel) to predicted proteins using FASTS (gray bars) and raw sequencing reads using TFASTS (white striped bars).

For each of the protein sequence databases described above (NM, CAFM, RFM, NM_KG, and RFM_KG), we concatenated the metagenomic protein predictions from multiple individuals into a single database. For example, NM, RM, RFM, and CAFM each contain metagenomic sequences from seven individual human samples from this study (15a, 15b, 16a, 16b, 18a, 18b, and 6b), which include an *unrelated* healthy sample 16b (Figure 2 comparisons). The NM_KG and RFM_KG protein databases contain the same 7 metagenomic predicted protein sequences (15a, 15b, 16a, 16b, 18a, 18b, and 6b), but unlike NM and RFM, contain the published 13 Japanese metagenome sequences [27] and 2 American metagenome sequences [26] for a total of 22 concatenated metagenomes per protein sequence database.

Deeper whole genome shotgun sequencing was obtained from an extra run on 6b and an additional sample (6a), extracted using the Zymo and MioBio method, which resulted in a four-fold increase in sequence data for these two healthy samples (Table S1). Due to the limitations of analyzing this larger metagenomic sequence dataset, these sequences were processed similar to the RM strategy and compiled into 2-independent protein databases, termed RMPS, for 6a and 6b in this assessment. Each of these 8 protein databases (NM, NM_KG, CAFM, RFM, RFM_KG, RM, RMPS-6a and RMPS-6b) included human reference sequences (July 2007 release, NCBI; ∼36,000 protein sequences) and common contaminants (i.e., trypsin and keratin; 36 protein sequences). Lastly, a 6-frame translation library was generated for sample 6a and searched against one MS experiment.

### Spectral Analysis

Microbial proteins were extracted and processed for 2D-LC-MS/MS as described [12] using an Ultimate HPLC system (Dionex, Sunnyvale, CA) coupled to a high resolution LTQ-Orbitrap (Thermo Fisher Scientific, San Jose, CA). Peptide mixtures from the two samples, 6a and 6b, were separated by a 12 step, multidimensional high-pressure liquid chromatographic elution profile consisting of eleven salt pulses followed by a 2 hr reverse-phase gradient from 100% solvent A (A: 95% H_2_O, 5% acetonitrile, 0.1% formic acid) to 50% solvent B (B: 30% H_2_O, 70% acetonitrile, 0.1% formic acid). Precursor full MS spectra (from 400–1700 *m/z*) were acquired in the Orbitrap with resolution = 30,000 followed by five data-dependent MS/MS scans at 35% normalized collision energy in the LTQ with dynamic exclusion enabled. All RAW files were converted to mzXMLs using ReAdW (v4.3.1; 2009) and mzXMLs subsequently converted to dta files using MzXML2Search (v4.3.1; 2009). All MS/MS were searched with SEQUEST (v.27) [2] for fully tryptic peptides (≤4 missed cleavages, 3 Da parent mass tolerance window, 0.5 Da fragment ion window) against each of the 8 custom-made FASTA formatted protein sequence databases described above. Since it is well established that trypsin cleaves primarily C-terminal to Arg and Lys [28], we have found in a variety of microbial communities [9], [29], [30] that using fully tryptic searches provides increased confidence in the peptide assignments while minimizing the potential for increased false positives due to incorrect candidate peptide sequences. All SEQUEST output files were assembled and filtered using DTASelect (v1.9) [31] at either a 2-peptide level for all seven: NM, NM_KG, RM, RFM_KG, RFM, CAFM, and RMPS databases and also 1-peptide level for the RMPS database searches with the following widely accepted parameters: cross correlation scores (XCorr) of at least 1.8, 2.5, 3.5 for +1, +2, and +3 charge states [9], [31], [32], respectively and a minimum deltCN of either 0.08 (default) for all seven databases (NM, NM_KG, RM, RFM_KG, RFM, CAFM, and RMPS databases) and/or 0.0 for NM, NM_KG, RFM, RFM_KG, RMPS-6a and -6b, and target-decoy databases (described under “false discovery rates”). Post-translational modifications and other fixed modifications were not included in the search criteria.

We used the high mass accuracy capabilities of the Orbitrap with a wide mass tolerance to measure precursor ion (peptides) masses at low parts-per-million (ppm) and the ion trap to efficiently measure fragment ions at lower resolution. A “post-database search” filter with high precursor mass accuracy was used by comparing the theoretically derived peptide from the SEQUEST mass with what was observed in the Orbitrap in the full scan preceding the MS/MS scan. Recently, Hsieh *et al*. [33] indicated that a wide precursor mass window in a database search and a post-database high precursor mass accuracy filter is a more superior method to control false positives. Therefore, for post-filtering the database results by high mass accuracy, the mass deviation (in ppm) of a PSM was calculated using the measured monoisotopic mass and theoretical monoisotopic mass of the peptide. For all of the database searches (NM, NM_KG, RM, CAFM, RFM, RFM_KG, RMPS-6b and -6a, and target-decoy databases) and comparisons, DTASelect was run with a t0 option to report all MS/MS spectra, in which case two spectra per protein, rather than two peptides, are required for identification. We compared each of the database results in a relative fashion such that all comparisons (degenerate peptides) are consistent to one another. Every MS/MS spectrum that is assigned to a peptide (unique and non-unique peptides) was noted and handled by DTASelect as described [31]; therefore, we recognize peptides that are shared (non-unique) among multiple proteins. While we recognize that non-unique peptides are somewhat problematic for label-free quantification using spectral counts, this was not the focus of the current study.

Spectral quality assessment was accomplished utilizing an in-house developed script that parses the SEQUEST output and mzXML formatted spectral data. All spectra collected during an analysis were categorized according to type: full MS scan (MS1) or tandem mass spectra (MS/MS). MS/MS spectra assigned to a peptide by SEQUEST were noted while the remaining unassigned MS/MS spectra were classified as high-quality or poor based on the following conditions: a. the charge state of the parent ion must be greater than 1, b. the minimum absolute intensity must be greater than 2500 counts, and c. greater than three fragment peaks within 20% of the based peak must be present (all other details in preparation to be submitted for publication). To quantify the peptide-spectrum success, MS/MS were categorized as (i) assigned or unassigned to a peptide and (ii) if unassigned, a score of high-quality or poor as reflected by four methods (NM, CAFM, RFM, and RMPS) and six databases (NM, CAFM, RFM, RFM_KG, and RMPS-6a and -6b).

All MS .raw files or other extracted formats and supporting information are available upon request. The acquired raw MS data associated with this manuscript may be downloaded from ProteomeCommons.org Tranche network through www.proteomecommons.org using the following hash: sI4rGyY9T4Uzd3eGfz+Jhj7W9MoB/YbrWEPLXNYd/tKi2wbaf+fP5fuDWRDbJuDrjf5FrunTjw0xWH2uPn0oXyAHrtUAAAAAAAAl3Q =  = .

### False Discovery Rates

A target-decoy database [34], [35] was generated for each of the five metagenomic processing methods (NM, CAFM, RM, RFM, RMPS), for a total of six forward-reverse databases (RM, RFM, CAFM, KG, NM_KG, and RMPS-6b) and searched against one of the two samples (6b) used in this study to estimate the peptide-level false discovery rate (FDR) with the new metagenomic processing methods. One sample and technical run (6b, Run1) was used to represent the entire sample set (2 samples; 4 runs) for each target-decoy database search in order to reduce the total number of target-decoy databases, search time, and complexity of comparisons. All target-decoy SEQUEST output files were assembled and filtered using DTASelect (v1.9) [31] with the same XCorr filters as described previously, and either a ≥1 peptide per protein with a deltCN filter of 0.0, or a ≥2 peptide per protein with a deltCN of 0.0 (RMPS-6b) or 0.08 (NM_KG, CAFM, RM, RFM, and KG), with an empirical FDR threshold of ≤2.0%. The initial, 1-peptide filter and deltCN 0.0, forward-reverse database searches provide FDRs for NM_KG, CAFM, RM, RFM, KG, and RMPS-6b (read-based) database analyses while the latter, 2-peptide and deltCN 0.08 filter, forward-reverse database searches contain the same filtering criteria as the original forward databases (NM, NM_KG, RM, RFM_KG, RFM, CAFM, and RMPS databases; Table 1 results) described earlier. Finally, a forward-reverse database was also created for the final paired metagenome sequence strategy (RMPS) for 6b and searched against the spectra collected from 6b, Run 1 and Run 2 using a deltCN 0.0, 1-peptide minimum, and high mass accuracy filtering. The identified peptides (both forward and reverse) were then mapped back to the protein sequences derived from the assembled metagenomic sequences using a post-database 2-peptide filter by exact string comparisons. Although the peptides with corresponding high mass accuracy measurements (±10 ppm) were considered for all downstream analyses, the peptide-level FDRs were estimated for both, with (−10≤ppm≤10) and without (ppm < -10 and ppm > 10) high mass accuracy, for 6b, Run1 against six genomic processing methods (NM_KG, CAFM, RM, RFM, KG, and RMPS-6b). Each protein entry (sequence) was reversed, i.e., the original N-terminus became the C-terminus. The new reverse (false) sequences were then appended onto the backend of the original forward sequences where each set, forward and reverse, represents 50% of the entire database. A peptide-level FDR was calculated based on the calculation: 2[n_rev_/(n_rev_ + n_real_)]*100 where n_rev_ is the number of peptides identified from the reverse database and n_real_ is the number of peptides identified from the real (forward) database [35].

### Sequences Similarity Searches

Peptides obtained from our SEQUEST/DTASelect searches were searched against the 6b and 16b protein databases using FASTS and against raw sequencing reads using TFASTS [36], using an e-value cutoff of 10^-5^.

### De novo Sequencing of Peptides by MS

PepNovo+ [37] and PEAKS [38] algorithms were used to *de novo* sequence MS/MS spectra collected from both samples, independent of all sequence databases. The PEAKS (v4.5 SP2) algorithm computes the best possible sequence among all probable amino acid combinations at a full peptide length confidence followed by individual amino acid confidence per residue in the predicted sequence for a MS/MS. PEAKS was run with default parameters with a parent mass error tolerance of 0.5 Da, fragment mass error tolerance of 0.5 Da, and trypsin digestion. First, a 90% confidence level was required for the overall, full length prediction to be correct and second, an 80% confidence level was required for each residue within that sequence, which is consistent with Ma *et al*. [38] PepNovo+ (v3.1) was executed using the following recommended parameters: -model CID_IT_TRYP -digest TRYPSIN -pm_tolerance 0.05 -num_solutions 5 -output_cum_probs. The top-scoring tags of all spectra were filtered using a cumulative probability cutoff of 0.5. In the sequence tags produced from both algorithms, the isobaric amino acid pair of Isoleucine (I) and Leucine (L) and the nearly isobaric pair of Lysine (K) and Glutamine (Q) are considered equivalent. L and I were both substituted with the letter, J, for convenience. Additionally, Q and K were substituted with the letter, U, since they are not easily resolvable (small mass difference of 0.036 Da) with ion trap MS/MS data. For all three algorithms, SEQUEST, PEAKS, and PepNovo+, a minimum of 3 residues has to be assigned to a spectrum for it to be considered for any additional analysis and comparison to other algorithms. For PEAKS, only the high confidence sequence tag was used for all analyses, not the predicted full-length peptide sequence. For the comparison of PSMs between all three algorithms, a “partial” consensus sequence was considered as a peptide sequence that has ≥3 amino acids that are exactly the same for the same mass spectrum between either SEQUEST peptide sequences, Peaks' high confidence sequence string, and/or Pepnovo+s' sequence tag. If a PSM has an “exact” consensus sequence with 100% sequence identity between any two or more algorithms, it would be considered a shared, exact consensus sequence. If a PSM does not have at least 3 residues within a peptide sequence string that match two or more algorithms, that spectrum would be considered unique to that algorithm. The identified SEQUEST/DTASelect PSMs for RMPS-6a and -6b sequence databases with a 1-peptide minimum and deltCN of 0.00 for 6a (Run 2 and 3) and 6b (Run 1 and 2) were compared to the PSMs from PEAKS and PepNovo+. The breakdown of partial and exact consensus sequences versus PSMs that are unique to a specific algorithm can be found in the Venn diagram. We did not take into account any single amino acid polymorphisms in the algorithms' consensus sequence comparisons. In this study, we controlled the false discovery rate by only using the high confidence consensus sequences tags found between the two *de novo* algorithms using their respective optimum parameters.

## Results

### Protein Sequence Database Comparison

Four protein prediction strategies (Fig. 1) were implemented for metagenomic DNA sequences obtained from two healthy human fecal samples (referred to as 6a and 6b), using a combination of assembly and gene prediction methods. Each protein sequence database has a defined acronym (2–4 letters), designating the strategy used (Fig. 1 and Table 1). Our goal was to increase peptide-spectrum matches using MS database searching for which the MS data was collected from the same samples as the DNA sequence data. The ability to accurately match peptides to tandem mass spectra (MS/MS) was assessed by comparing the number of PSMs and unique peptides identified for each database search with SEQUEST/DTASelect at a 2-peptide level, deltCN 0.08, and XCorr filtering against the same 2 samples, 6a (with spectra from runs 2 and 3) and 6b (with spectra from runs 1 and 2) (Table 1). These results illustrate how common metagenomic processing methods (assembly and ORF finding) affect peptide and spectra identification (Table 1). From these results, three major trends emerge: (A) Collapsing of the sequence data by assembly decreases the number of assigned spectra. There was a decrease of assigned spectra when all reads were assembled from all samples compared to assembly by individual sample (NM, 23,026 spectra vs. CAFM, 17,470 spectra). Additionally, if reads are annotated without assembly, PSMs increase (NM, 23,026 spectra vs. RM, 34,666 spectra). This can be largely attributed to the increased diversity of possible peptides, determined by *in silico* trypsin digestion, in the unassembled data, which is over 3 times what is found in assembled data (5,638,100 vs. 1,639,802). (B) An increase in spectrum assignment usually translates to an increase in unique peptide identifications. For example, the 11,640 gains in spectral assignment translate to a 3,624 gain in identification of unique peptide sequences for RM compared to NM (Table 1). However, this was not observed when comparing CAFM to NM, where the 5,556 gains in spectra assignment translated to a decrease of 2,638 unique peptides (Table 1). (C) *De novo* gene finding methods are sufficient for optimal spectrum assignment. The combined *de novo* and homology-based gene finding method did not increase PSMs as hypothesized (RFM, 34,665 spectra vs. RM, 34,666 spectra) nor the number of identified unique peptides (RFM, 9,608 peptides vs. RM, 9,618 peptides; Table 1).

Because of the low relative sequence coverage of our metagenomic samples, we wanted to evaluate whether adding metagenomic sequences from 15 unrelated samples in two published studies would enhance our spectrum assignment. Therefore, to protein databases NM and RFM, we added the proteins sequences from predicted ORFs from two published human gut metagenomic studies, referred to as “KG” for Kurokawa *et al*. and Gill *et al*. [26], [27], which are referred to as NM_KG and RFM_KG respectively. The KG database contains 13 metagenomes from a Japanese cohort [27] and 2 metagenomes from an American cohort [26], both geographically distinct from samples in this study. When compared to the metagenomic sequences in this study, only 9% of sequences align in KG at 99% identity or greater; thus, they provide over 2 million additional unique peptides for MS/MS assignment, that are not identified in any of the matched metagenomes. Because the assemblies from these studies are on average longer (average contig length of 2,300 nt for Kurokawa *et al.* compared to an average contig length of 1,128 nt in this study), the predicted proteins are more likely to be full-length compared to ORFs in this study (average protein length of 194.5 amino acids (aa) for Kurokawa *et al*. metagenomes; average protein length of 225 aa for Gill *et al*. metagenomes compared to an average protein length of 168.5 aa in this study). By including metagenomic sequence from additional sources [26], [27], the number of identified spectra increased (NM versus NM_KG (23,026 versus 41,267 spectra) and RFM versus RFM_KG (34,665 versus 43,726 spectra)) for 6a (Run 2 and 3) and 6b (Run 1 and 2) in total (Table 1). However, the additional KG sequence data came at the cost of increased *peptide degeneracy* and subsequent *protein redundancy* (i.e., peptides mapping to multiple proteins or to the same protein in multiple metagenomes within the sequence database). Although the level of redundancy ranges with the sequence diversity of a sample and has no effect on the actual database search algorithms, this complicates protein inference and assigning its' corresponding phylogenetic origin in a complex environmental community.

While the four metagenomic processing methods were compared based on their ability to comprehensively assign all collected MS/MS spectra to peptides, the percentage of *assigned* and high-quality *unassigned* MS/MS is equally important to establish the utility of each sequence database. For the following spectral analyses, the collected and assigned spectra from sample 6a (Run 2 and 3) and 6b (Run 1 and 2) were assessed and categorized after applying the same filters described above (2-peptide level and deltCN 0.08 filter) with the following databases. Of the total MS/MS collected during one MS experiment (70,000–81,000), on average 6,600 spectra were assigned to a peptide sequence in the NM database (∼8% of total collected MS/MS spectra for a single run; Table S2). In contrast, the processing strategy used to create RFM resulted in the assignment of an additional 1,800 MS/MS from the same sample, for a total of 8,430 peptide-spectrum matches on average (11% of total collected MS/MS). Furthermore, the addition of unrelated KG sequences to RFM (a 25% increase in sequence data) resulted in an increase of the number of assigned spectra by only 2–3%. Finally, the strategy used to create RMPS resulted in an additional 4,000 MS/MS spectra assigned, for a total of 12,461 peptide-spectrum matches on average per sample (16% of total collected MS/MS spectra). Although the total number of assigned MS/MS increased from NM < RFM < RFM_KG < RMPS, the number of unassigned, high-quality spectra decreased with database quality (NM > RFM > RFM_KG > RMPS).

The effects of two common filtering parameters (deltCN and high mass accuracy) on MS/MS peptide assignment were examined by determining the quantity of MS/MS spectra not assigned to the same peptide in multiple database searches (Text S1). These results (Figure S1) suggest that filtering on high mass accuracy rather than deltCN can decrease ambiguous peptide-spectrum matches and provide more consistent and reproducible MS/MS identifications. In order to maintain high specificity and accuracy with increasing metagenomic sequence data, a FDR was estimated at the peptide level using an established method of reverse database searching [34], [35] for each metagenomic processing method for a total of 6 target-decoy databases (RM, RFM, CAFM, KG, NM_KG, RMPS-6b). Because we are using methods that directly measure peptides, not proteins, the FDR was estimated at the peptide level. In addition, we are primarily comparing the performance of all databases by peptide-spectrum matches, not proteins, given the nature of the metagenomic processing methods and their corresponding databases (i.e., not all databases contain assembled contigs, but only reads). It has previously been noted [39] that false discovery rates can be difficult to accurately determine with metaproteome datasets due to problems associated with massive peptide degeneracy. We concur with this difficulty in accurately quantifying FDRs for metaproteomes and thus have carefully evaluated how we might handle this issue, as defined in the following discussion. In this study, for example, of all the identified peptides for 6a (Run 2), only 7–30% were unique peptides from each database. Consequently, if only unique peptides are used, the false discovery rate would be overestimated; on the contrary, if all peptides are used the false discovery rate could be underestimated [39]. Therefore, to set a static FDR threshold and filter multiple databases (6 sequence databases in this study) of different sizes and internal levels of peptide redundancy to that threshold (i.e., 1%) becomes a challenge, in this case, for comparing and identifying the best metagenomic processing method for MS/MS database searching and peptide-spectrum matching. As the level of redundancy affects the FDR, we have chosen a set of fixed scoring filters in order to accurately compare database assignments. Thus, the same filter criteria (i.e., Xcorr and ppm filtering) was applied to all database searches and with a requirement that the FDR be less than or equal to, i.e., 2.0%. The FDRs for the 1-peptide level, deltCN 0.0, with and without HM filtering were 1.17%–2.03% and 16.09–31.47%, respectively for 6b, Run 1 (Table S3). The 2-peptide level and deltCN 0.08 filtered reverse database searches serve to represent the FDR of peptide identifications found in Table 1. The FDRs for these PSMs, with and without HM filtering were within 0.09%–0.38% and 2.17–4.15%, respectively for 6b, Run 1 (Table S4). Following the application of a post-database high precursor mass accuracy filter (± 10 ppm) to both, the 1- and 2-peptide filtered forward-reverse datasets, the number of identified reverse peptides decreased by, on average, 93% for each database which resulted in a reduction of the FDR to 0.09%–0.38%.

### Tracking Missing Peptides

By adding the unrelated KG metagenomic sequences to the RFM protein database, the number of additional predicted unique peptide sequences increased by 40%. Therefore, we wanted to determine how many additional peptide-spectrum matches were gained by adding these KG proteins sequences to the database. The RFM_KG assigned MS/MS were distributed into three different categories: RFM only, KG only, and RFM plus KG (shared) for each sample (Table S5 and Text S1). The majority of RFM_KG assigned spectra were “shared” between both RFM and KG protein sequences. About 26% of the total spectrum assignments were unique to the RFM protein sequences (zero overlap with KG sequences) and only ∼8% of the spectra were unique to the KG protein sequences (no overlap with the RFM sequences) (Table S5).

There are two possible hypotheses for why the metagenomes from these samples (i.e., RFM) cannot be used to assign peptides to spectra which are assignable by the unrelated protein database KG: (1) because of low sequencing depth, peptides are not assigned because our protein database is incomplete or (2) because of a sequencing error or limitation for predicting ORFs, we are unable to predict the proteins that are present. Therefore, we have aligned the RFM_KG (2-peptide, deltCN 0.08, HM filtered) *identified* peptides (Fig. 2, y-axis) from 6a (left panel) or 6b (right panel) to *predicted* raw reads from the related/same sample (6b) and an *unrelated* sample (16b) (Fig. 2, x-axis) using TFASTS [36] (Fig. 2, white, fine striped bars). Those results were compared to alignments of the same identified peptides to the predicted protein database from the related/same sample (6b) and the unrelated sample (16b) using FASTS [36] (Fig. 2, gray, solid bars). As expected, more peptides mapped to the related/same (matched metagenome-metaproteome) sample (15% for 6a: left panel, Fig. 2 and 6b: right panel, Fig. 2) than to the unrelated, 16b, predicted protein sequences (8% for 6a and 10% for 6b). When these same peptides were compared using TFASTS [36] (algorithm that compares peptides to DNA sequence) to the raw sequencing reads (Fig. 2, white, fine striped bars), the number of peptides matching to reads increased by two-fold for both 6a and 6b.

**Figure 3:**
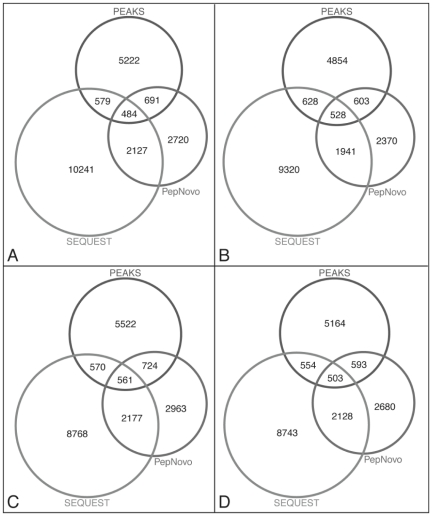
Performance and comparison of *de novo* peptide sequencing results. Distribution of assigned spectra per *de novo* algorithm with a predicted consensus sequence (partial and/or exact sequence match) among all three algorithms, PEAKS, PepNovo+, and SEQUEST. Identified peptides from SEQUEST and RMPS sequence database were compared to the *de novo* predicted peptides for (A) 6a Run 2, (B) 6a Run 3, (C) 6b Run 1, and (D) 6b Run 2.

### Targeting Peptide Discovery

Throughout the course of our study, we were able to accumulate more metagenomic sequence data for the two healthy samples, 6a and 6b, by ∼5 fold (Table S1, italicized text). Although this increase in predicted ORFs resulted in an increase in the number of assigned MS/MS spectra, it can reduce the throughput of MS/MS sequence-database searching. Therefore, we investigated the impact of searching a metagenomic-based protein database derived from the exact same single sample to that of a concatenated sequence library of all available metagenomic data from this study. The additional metagenomic sequences were used to construct a sequence database similar to that of RM (non-assembled reads with 5.6 million predicted unique peptides), called RMPS (Fig. 1) which has ∼ 1.3 million predicted unique peptides, on average, per healthy sample 6a and 6b. Searching the RMPS sequence databases with SEQUEST using standard 2-peptide, deltCN 0.08, and high mass accuracy filtering decreased the compute time to ∼300–500 minutes per MS raw file. By increasing the amount of metagenomic sequence data for a single sample, the total number of assigned spectra increased by 63% (from 34,666 to 56,782) and the number of total identified non-redundant (NR) peptides increased by 67% (from 9,618 to 16,055) (Table 1, RM versus RMPS), resulting in a 54% increase in protein identifications (3,394 to 5,233) when mapping these peptides to a protein dataset generated from assembled reads for the *exact same* metagenomic sample.

Other than limitations associated with computational resources, there was also a concern that real peptides predicted from 454-reads would be filtered out given a 2-peptide per protein minimum filter (Table 2, top panel). Therefore, the filtering parameters were readjusted with a deltCN 0.0, 1-peptide minimum, and a high mass accuracy filter (±10 ppm) for the SEQUEST RMPS database searches for both 6a (Run 2 and 3) and 6b (Run 1 and 2). The identified peptides were then mapped back to the predicted protein sequences derived from the assembled metagenomic sequences with a 2-peptide filter, resulting in an increase of protein identifications, from 5,233 to 6,186 (Table 2, RMPS top panel versus bottom panel). The filtering parameters were also readjusted with a deltCN 0.0 and a high mass accuracy filter (±10 ppm) for the SEQUEST-RFM database searches for both 6a (Run 2 and 3) and 6b (Run 1 and 2). The protein identifications also increased, from 3,431 to 3,706 (Table 2, RFM top versus bottom panel). While this increase might seem minimal, there is significantly less redundancy, less false positives, and no computational cost added to these filtering parameters. The false discovery rate, using the same filtering parameters (deltCN 0.0, 1-peptide minimum and HM) for the RMPS database was 1.17% for 6b (Table S3), however, these identified peptides (≥1 peptide/*read*) were mapped back to the predicted protein sequences derived from the assembled metagenomic sequences using a post-database ≥2-peptide/*protein* filter. Following application of this 2-peptide/*protein* filter, the FDRs dropped to 0.1%–0.2% for 6b, Run 1 and 2 (Table S6).

**Table 2:** Comparison of RFM and RMPS database results with different filtering metrics and a post-database mapping strategy.

Protein Database	RFM		RMPS	
**2-peptide, deltCN 0.08, HM Filter**				
	Spectra	Protein	Spectra	Protein
6a Run 2	3,246	1,154	6,542	1,761
6a Run 3	3,091	1,010	6,237	1,544
6b Run 1	2,639	637	5,212	973
6b Run 2	2,552	630	4,870	955
**Total**	11,528	3,431	22,861	5,233
**1- or 2-peptide, deltCN 0.0, HM Filter**				
	Spectra	Protein	Spectra	Protein
**Peptide Criteria**	≥2 peptide		≥1 peptide	
6a Run 2	3,541	1,252	7,497	2,069
6a Run 3	3,346	1,088	7,048	1,808
6b Run 1	2,879	686	5,881	1,182
6b Run 2	2,786	680	5,502	1,127
**Total**	12,552	3,706	25,928	6,186

Comparison of SEQUEST/DTASelect database search results, non-redundant spectra and protein counts with different filtering parameters and HM, post-database mapping of identified peptides to a protein dataset generated from assembled reads for the same metagenomic sample.

### De novo Peptide Sequencing

Two popular algorithms, PepNovo+ [37] and PEAKS [38], were used to identify peptide sequences *de novo* from MS/MS spectra collected from both samples, independent of all protein sequence databases. Initially, the two algorithms were run independently on the same raw MS data and samples as described. The identified, high confidence consensus sequence tags (≥3 residues) were acquired from each *de novo* algorithm. The *de novo* consensus sequence tags (Text S1) for PEAKS and Pepnovo+ were compared for every MS/MS to identify the partial (≥3 residues) and exact consensus sequence tags that would represent the most confident PSMs identified by the two different *de novo* algorithms. In this study, it was not our goal to compare the performance of the two programs; instead, we want to combine the best results from the two programs using their respective optimum parameters. The final, representative *de novo* consensus tags were compared to the previously mentioned SEQUEST results from the RMPS sequence database searches that were filtered at a ≥1 peptide/read, deltCN 0.0, and high mass accuracy with a post-database ≥2 peptide/protein filters. On average, ∼593–724 MS/MS spectra were assigned with a high confidence consensus peptide sequence between the two *de novo* algorithms, but were not assigned with the SEQUEST–RMPS database search (Fig. 3). These *de novo* peptide sequences were mapped to protein sequences predicted from assembled contigs with a 2-peptide minimum per protein and compared to the peptides that were identified from the SEQUEST-RMPS database searches. A total of 421 new, non-redundant proteins were identified with the *de novo* sequenced peptides for metagenome 6b, and 333 non-redundant proteins for metagenome 6a; these proteins were not identified using SEQUEST. Approximately 450 *de novo* sequenced peptides (non-redundant) per sample could not be mapped to the matched metagenomic sequence data.

## Discussion

One of the major goals of MS-based proteomics is to comprehensively identify the protein complement of a given sample (isolate, mixture, or community). The proteome(s) of microbial communities are highly complex and pose numerous challenges for MS experimentation and analysis. These challenges include the dynamic range of peptide abundances and a number of informatics hurdles, such as differentiation between closely related species, identification of sequence polymorphisms, and global identification of post-translational modifications. Many of the algorithms used in MS/MS database searching are based on the assumption that a protein is derived from a single organism with little sequence diversity. However, these assumptions are no longer valid in the case of complex microbial communities. This study presents several strategies for improving metagenomic guided MS-based metaproteomic peptide-spectrum matching in complex samples.

It has become very clear that the quality of metagenomic sequence data and resulting protein sequence database has a significant impact on community MS-based proteomics and the ability to achieve deep proteome coverage. This study initially explored how assembly and gene finding methods for metagenomic sequences affects peptide-spectrum matching. Our findings suggest that predicting ORFs from an *ab-initio* gene finder on metagenomic reads provides the best database for maximal MS/MS assignment. While assembly of metagenomic data can greatly reduce the necessary compute time for gene finding and database searching, it essentially collapses sequence diversity; thus, it is sub-optimal for maximal spectral assignment. Yet, introducing a homology-based gene finding method (RFM) does not increase the number of assigned spectra. Lastly, with an increase in sequence coverage for a biological sample, our results suggest that predicted protein sequence databases derived from matched metagenomic sequenced reads (RMPS), increases the number of MS/MS spectra, peptides, and protein identifications. In conclusion, expanding the metagenomic sequence library for matched or related samples improved peptide-spectrum matching. However, improvements in gene finding are equally important to maximize protein identification and coverage.

As the matched metagenomic predicted protein sequence database (RMPS) more accurately reflected the “true proteome”, previously unassigned high-quality spectra are now being identified and provided greater proteomic depth. When these results were compared to a standard bacterial isolate (e.g., *E. coli*) with a well-curated genome, ∼41,000 MS/MS spectra were assigned to peptides (37% of total collected MS/MS) (data not shown) using the same database searching filters (≥2 peptide and deltCN 0.08). This would suggest that underlying challenges are still inhibiting the identification of a majority of spectra collected from the community samples compared to that of a standard bacterial isolate. The classification of acquired and assigned MS/MS spectra and quantification of total identified peptides suggested that the RMPS processing method provided the most comprehensive assignment of MS/MS spectra.

When we examine why some peptides are assigned from the read-based ORFs (e.g., RMPS processing method) and not assigned from the contig-based ORFs (e.g., NM processing method), we find that these “lost peptides” fall into three categories: (i) some reads are not assembled and therefore their protein predictions are not in the contig-based ORF predictions, (ii) because of SNPs and frameshifts, the peptides are 100% similar to a predicted contig-based ORF, but are not 100% identical, and (iii) some peptides were very different (<50% identical) or missing from the contig-predicted ORF. A 6-frame translation protein database was generated for sample 6a to capture all possible candidate peptide sequences and searched against one MS experiment (Run 2). However, routine use of this sequence database is impractical due to the increased quantity of sequences which directly correlates with an increased quantity of candidate peptides, therefore, more scoring and prohibitively large search times (∼134 hrs per MS experiment) (data not shown). As sequencing data generation increases, even a read-based strategy could become unsustainable, which will only worsen as new larger ‘omic’ datasets become available.

Identifying the most reliable set of peptides from a MS-based metaproteomic experiment can be complicated, as we have shown that MS/MS assignments can vary and be assigned to different peptide sequences with different protein databases. While filtering on deltCN is a common practice for reducing false positives, this type of filtering may (i) continue to include many ambiguous peptides based on the different database predictions and (ii) remove many legitimate peptides as a result of a highly redundant database. Although filtering on deltCN and peptide-protein matches has proven effective for single genome searching, these filters decrease both precision and sensitivity in metagenomic predicted sequence databases. As common filtering strategies have proven to be less effective and practical for large-scale proteomics studies (e.g., post-translational studies) [33], these and other challenges will surface as the MS field moves towards sampling more environmental communities. Alternatively, we propose that when high mass accuracy is used in conjunction with other filtering metrics, such as, cross correlation (XCorr) and enzyme cleavage specificity, one can confidently identify the most comprehensive and reproducible set of PSMs and control false positives adequately in a complex environmental community sample. As shown, this strategy greatly reduces the rate of ambiguous peptide predictions thereby giving higher confidence to our final peptide-protein identifications. Once peptides are identified and mapped to metagenomic sequences, which have been assembled, the subsequent use of a 2-peptide filter greatly reduces the number of false positives in protein discovery for complex microbial environments.

Finally, *de novo* peptide sequencing can complement MS/MS database searching to identify peptides absent in the protein sequence database due to the limitations of the gene finding algorithms or low metagenomic sequence coverage. We believe that novel peptides were identified with high confidence in this study, because these peptides were independently identified by two *de novo* sequencing algorithms. However, there is no widely accepted method for us to use for rigorously evaluating the FDRs of novel peptides identified from our microbial community samples. Thus, *de novo* sequencing results should be used with the caveat of uncertain FDRs as supplement to database searching results [40].

By using a variety of MS filtering metrics, we were able to assess the quality and accuracy of MS/MS peptide sequencing for each MS experiment against four predicted protein sequence databases derived from whole genome shotgun sequences. Our findings suggest that: (i) proteomic data is twice as likely to match metagenomic data derived from the same sample, (ii) although unrelated metagenomic data may capture more sequence diversity, large protein databases can create unreasonable sequence redundancy, thereby hampering the ability to differentiate real peptide-protein identifications, (iii) the percentage of unassigned, high-quality MS/MS spectra decreases with increased quality of metagenomic sequences, (iv) metagenomic data processing, such as assembly and gene finding, affects the ability to assign peptides to spectra, (v) MS filtering metrics can affect the accuracy of peptide-spectrum matching, (vi) deeper metagenomic sequencing coverage results in deeper coverage of matched metaproteomes and (vii) *de novo* peptide sequencing can overcome potential sequencing errors and provide evidence for novel sequences not yet sequenced or not identified by database searching methods. The high-quality unassigned MS/MS from sequence-database searching would be ideal target spectra to submit for *de novo* peptide sequencing whereby these sequences could be mapped back to help refine the metagenome and identify potential sequencing errors. Finally, this study illustrates how common metagenomic processing methods (assembly and ORF finding) and database construction can affect metaproteomics search results.

## Supporting Information

Figure S1 Accuracy Assessment by DTASelect Filtering. (a) For each DTASelect peptide prediction search, the number of identified spectra was calculated and compared using three different parameter combinations, deltCN filtered results at a deltCN of 0.08 only, both deltCN of 0.08 and HM (±10 ppm), and HM (±10 ppm) only, where identified peptide sequences were designated either ‘Consistent’ (solid gray) or ‘Inconsistent’ (diagonal stripes). (b) A VENN diagram with assignable spectra for RFM, RFM_KG, NM, and NM_KG databases, filtered by high mass accuracy, for both samples combined.
(EPS)

Table S1 Metagenomic sequencing metrics.
(XLS)

Table S2 Database dependent distribution of acquired full MS and MS/MS and assigned MS/MS for samples 6a and 6b. Unassigned MS/MS were parsed into either quality or poor spectra.
(XLS)

Table S3 False discovery rates for sample 6b (Run 1) against six different metagenomic-predicted sequence databases. The database results were filtered at a 1-peptide level with and without high mass accuracy.
(XLS)

Table S4 False discovery rates for sample 6b (Run 1) against six different metagenomic-predicted sequence databases. The database results were filtered at a 2-peptide level with and without high mass accuracy.
(XLS)

Table S5 Distribution of RFM_KG assigned PSMs for 6a (Run 2 and 3) and 6b (Run1 and 2). The assigned PSMs were distributed into three different categories: RFM only, KG only, and RFM plus KG based on their sequence uniqueness to each set of sequences. If a PSM was unique to protein sequences in RFM, but was not present in KG, the PSM was classified and categorized as RFM only and vice versa. If a PSM was found to match a protein in both, RFM and KG, the PSM was categorized as a shared spectrum.
(XLS)

Table S6 False discovery rates for sample 6b (Run 1 and 2) against the RMPS database. An initial ≥1-peptide, deltCN 0.0, and high mass accuracy (±10 ppm) filter were applied to the read-based identifications followed by a ≥2-peptide/protein post-database mapping filter.
(XLS)

Text S1 Additional supporting information and results for the protein sequence database comparisons, tracking missing peptides, and *de novo* peptide sequencing.
(DOC)
