# Supplementary material for: Strategies for Metagenomic-Guided Whole-Community Proteomics of Complex Microbial Environments
Source: PLoS One. 2011 Nov 23;6(11):e27173. doi: 10.1371/journal.pone.0027173 (PMC3223167; doi:10.1371/journal.pone.0027173)
Supplement: Text S1 — Additional supporting information and results for the protein sequence database comparisons, tracking missing peptides, and de novo peptide sequencing. (DOC) [file pone.0027173.s008.doc]

**Supporting Information (Text S1)**

**Protein Sequence Database Comparison**

Database searching algorithms and pipelines that aim to combine traditional filtering criteria and scores are somewhat problematic for metaproteomic datasets. We have found that deltCN filtering, one of many ways to filter MS/MS spectra, can be less effective for community metaproteomics data because of: 1) peptide redundancy in microbial communities (not only between multiple proteins, but also species/strains), i.e., certain peptides seem to be unfairly punished because of many very similar peptide sequences in the database, 2) assigned score ambiguities and 3) loss of correct PSMs. The effects of two common filtering parameters (deltCN and high mass accuracy) on MS/MS peptide assignment were examined by determining the quantity of MS/MS spectra not assigned to the same peptide in multiple database searches. A spectrum *not assigned* to the same peptide across different databases (NM, NM_KG, RFM and RFM_KG) was considered as an ambiguous assignment (inconsistent). The use of a default deltCN (which discriminates between true and false PSMs) value of 0.08 led to an ambiguous peptide assignment for 5,479 MS/MS spectra from sample 6a (Run 2 and 3) and 6b (Run 1 and 2). When filtering on high mass accuracy (HM) (±10 ppm) instead, this number decreased to 1,962 ambiguous spectra. Therefore, a deltCN filter of 0.08 resulted in ambiguous predictions for 7.8% of the total assignable spectra, in contrast with HM filtering where only 3.7% of assigned spectra were ambiguous. Surprisingly, when HM and deltCN (HM.deltCN) filters are both applied, 1,592 (3.1%) of the total assigned spectra have ambiguous assignments (Fig. S1a).

Even though each predicted protein sequence database is generated from the same metagenomic sequences, the percent of MS/MS that are assigned to a peptide varies depending on the metagenomic processing method, i.e. assembly and gene finding. Of the total assigned MS/MS from sample 6a, Run 2 and 3, and 6b, Run 1 and 2, for protein sequence database NM, NM_KG, RFM and RFM_KG (≥2 peptide and deltCN 0.08 filter), only 45% of the spectra had a peptide-spectrum assignment in all four databases (regardless of whether the spectrum had the same peptide assignment) when a HM filter was applied (Fig. S1b). The remaining 55% were spectra that were uniquely assigned to only one of the four databases. Additionally, of the total assigned MS/MS, ~18% of the spectra were assigned peptide sequences derived from the *unrelated* KG protein sequences in the NM_KG and RFM_KG databases. Because RFM is a subset of the protein sequences in RFM_KG, we expected all MS/MS assigned with the RFM database to also be assigned peptides in the RFM_KG database. However, ~ 1% of the total assigned MS/MS were unique to RFM (not assigned with RFM_KG). A similar result was also seen with the NM versus NM_KG spectra comparisons, ~ 2% of the assigned MS/MS were not assigned with the NM_KG sequence database.

**Tracking Missing Peptides**

In order to determine whether any peptides were absent in the related and matched metagenomes relative to the unrelated KG metagenomes, the RFM_KG assigned tandem mass spectra (≥2 peptide and deltCN 0.08 filter) were distributed into three different categories (RFM only, KG only, and RFM and KG; Table S5). If a PSM was unique to protein sequences belonging to RFM that was not present in the KG sequences, the spectrum would be considered unique and categorized as “RFM only” and vice versa for “KG Only.” However, if a PSM matched to both, protein sequences in RFM and KG, this spectrum would be considered a “shared spectrum” and categorized as “RFM and KG.” To note, spectra assigned to the human protein sequences and common contaminants were excluded from this analysis with the exception that they are included in the “total assigned spectra” column of Table S5.

***De novo* peptide sequencing**

The distribution of high confidence sequence lengths for all 4 runs with PEAKS was 9 aa (distribution of consensus sequence lengths is 3-47 aa) while the full peptide length average for all 4 runs was 22 aa. The latter full-peptide predictions were not included in any further analyses; only the high confidence sequence tags were used further to represent PEAKS PSMs. Pepnovo+ also had a sequence tag average length of 9 aa where the distribution of consensus sequence lengths is between 7-16 aa for all 4 runs.
